# *Leishmania donovani* visceral leishmaniasis diagnosed by metagenomics next-generation sequencing in an infant with acute lymphoblastic leukemia: a case report

**DOI:** 10.3389/fpubh.2023.1197149

**Published:** 2023-06-22

**Authors:** Li Chang, Guanglu Che, Qiuxia Yang, Shuyu Lai, Jie Teng, Jiaxin Duan, Ting Liu, Fang Liu

**Affiliations:** ^1^Department of Laboratory Medicine, West China Second University Hospital, Sichuan University, Chengdu, Sichuan, China; ^2^Key Laboratory of Birth Defects and Related Diseases of Women and Children, Sichuan University, Ministry of Education, Chengdu, Sichuan, China

**Keywords:** visceral leishmaniasis, *Leishmania donovani*, metagenomic next-generation sequencing, acute lymphoblastic leukemia, rapid diagnosis, case report

## Abstract

**Background:**

Visceral leishmaniasis (VL) is a neglected vector-borne tropical disease caused by *Leishmania donovani* (*L. donovani*) and *Leishmania infantum* (*L. infantum*). Due to the very small dimensions of the protozoa impounded within blood cells and reticuloendothelial structure, diagnosing VL remains challenging.

**Case presentation:**

Herein, we reported a case of VL in a 17-month-old boy with acute lymphoblastic leukemia (ALL). The patient was admitted to West China Second University Hospital, Sichuan University, due to repeated fever after chemotherapy. After admission, chemotherapy-related bone marrow suppression and infection were suspected based on clinical symptoms and laboratory test results. However, there was no growth in the conventional peripheral blood culture, and the patient was unresponsive to routine antibiotics. Metagenomics next-generation sequencing (mNGS) of peripheral blood identified *196123 L. donovani* reads, followed by *Leishmania* spp amastigotes using cytomorphology examination of the bone marrow specimen. The patient was given pentavalent antimonials as parasite-resistant therapy for 10 days. After the initial treatment, *356 L. donovani* reads were still found in peripheral blood by mNGS. Subsequently, the anti-leishmanial drug amphotericin B was administrated as rescue therapy, and the patient was discharged after a clinical cure.

**Conclusion:**

Our results indicated that leishmaniasis still exists in China. Unbiased mNGS provided a clinically actionable diagnosis of a specific infectious disease from an uncommon pathogen that eluded conventional testing.

## Introduction

Visceral leishmaniasis (VL) is a vector-borne protozoan neglected tropical disease (NTDs) caused by *Leishmania donovani* complex (*L. donovani* and *L. infantum*) and *L. donovani* (1–3). It is caused by an infection of blood cells in the lymphoid organs, primarily the spleen, bone marrow, and liver, and is fatal in more than 95% of untreated cases (4). In China, 3,169 cases of VL have been reported, with ~140–509 cases diagnosed per year between 2002 and 2011. VL is considered endemic in over 50 counties across 6 provinces/autonomous regions in western China, including Xinjiang, Gansu, Sichuan, Shaanxi, Shanxi, and Inner Mongolia (5–8). According to these data, leishmaniasis is not extinct and could potentially cause a public health problem in China.

Acute lymphoblastic leukemia (ALL) is the most frequent type of pediatric cancer, with an incidence of 5.4 per 100,000 cases in patients aged < 15 years old (9). In addition, cases of leishmaniasis found in patients formerly diagnosed with various cancers and treated with long-term anti-cancer chemotherapy have been previously reported, clearly suggesting an overlap between leishmaniasis transmission and malignant disease (10).

The diagnosis of VL is based on detecting *Leishmania* amastigote parasites in bone marrow or spleen biopsies. However, the very small dimensions of the protozoa impounded within blood cells and reticuloendothelial structure makes diagnosing leishmaniasis challenging (11). Recently developed metagenomics next-generation sequencing (mNGS) analyses forego the use of specific primers or probes. Instead, the entirety of the DNA and/or RNA (after reverse transcription to cDNA) is sequenced, thus providing a practical approach for diagnosing rare, novel, and atypical infectious etiologies (12). In the following description, we reported a VL case in an ALL infant after chemotherapy diagnosed by mNGS and parasitological microscopy.

## Case presentation

A 17-month-old boy was admitted to West China Second University Hospital, Sichuan University, in March of 2022 for the insidious onset of fever, ecchymosis of skin, anhelation, and pancytopenia. On admission, blood routine examination results were as follows: white blood cell (WBC) 2.5 × 10^9^/L, hemoglobin (Hb) 60 g/L, platelet (PLT) 45 × 10^9^/L, and immature granulocytes found in peripheral blood smears. Subsequently, bone marrow morphology, immunophenotyping, cytogenetics, and molecular genetics were carried out. Based on the above testing, the boy was diagnosed with B-cell acute lymphoblastic leukemia (B-ALL, L2, ETV6-PEX5 fusion gene positive, KRAS A146V and KRAS A146T mutation, IKZF1–8 heterozygous deletion, 45, XY, der (7; 12) (q10; q10)(5)/46, XY (15). Then, according to ALL-low risk (ALL-LR) of the Chinese Children's Cancer Group study ALL 2020 (CCCG-ALL-2020), conventional and continuous therapy was administered. After the remission induction regimen of 4 weeks, complete remission (CR) was reached. Subsequently, the child was supposed to receive consolidation therapy according to CCCG-ALL-2020 with three courses after 4 weeks of achievement of CR.

On October 2022, which was also the interval between the first consolidation treatments, the patient was hospitalized again for a repeated fever of 13 days and coagulopathy after the second cycle of consolidation chemotherapy. Laboratory tests are shown in Table 1. Color Doppler ultrasonic examination showed swollen liver and spleen. The above results suggested that the infant had chemotherapy-related bone marrow suppression, infection, and coagulation dysfunction. Vancomycin, imipenem, and voriconazole were used for empirical antibiotic therapy. Fresh frozen plasma, fibrinogen, and prothrombin complex were used for improving coagulation function. Seven days after therapy, the patient still had a fever (38.4–39.4°C), and his liver and spleen were enlarged. CRP and PCT levels were 125.5 mg/L and 3.99 mg/L, respectively (Table 1). No microorganisms were detected by blood culture.

**Table 1:** Laboratory test results of a patient during diagnosis and treatment.

**Enrollment time**	**WBC ( × 10^9^/L)**	**N (%)**	**L (%)**	**E (%)**	**Hb (g/L)**	**PLT ( × 10^9^/L)**	**PT (S)**	**APTT (S)**	**Fg (mg/dL)**	**CRP (mg/L)**
On admission	1.0	60.4	29.5	0	85	8	15.5	43.6	105	76.9
7 days after antibiotic treatment	12.2	91.0	3.0	0.1	122	39	12.7	34.2	200	125.5
10 days after sodium stibogluconate treatment	1.5	74.7	9.3	0	81	159	10.7	25.4	202	7.2
14 days after Amphotericin B	2.8	39.1	35.0	0.2	110	182	/	/	/	0.5

**Figure 1:**
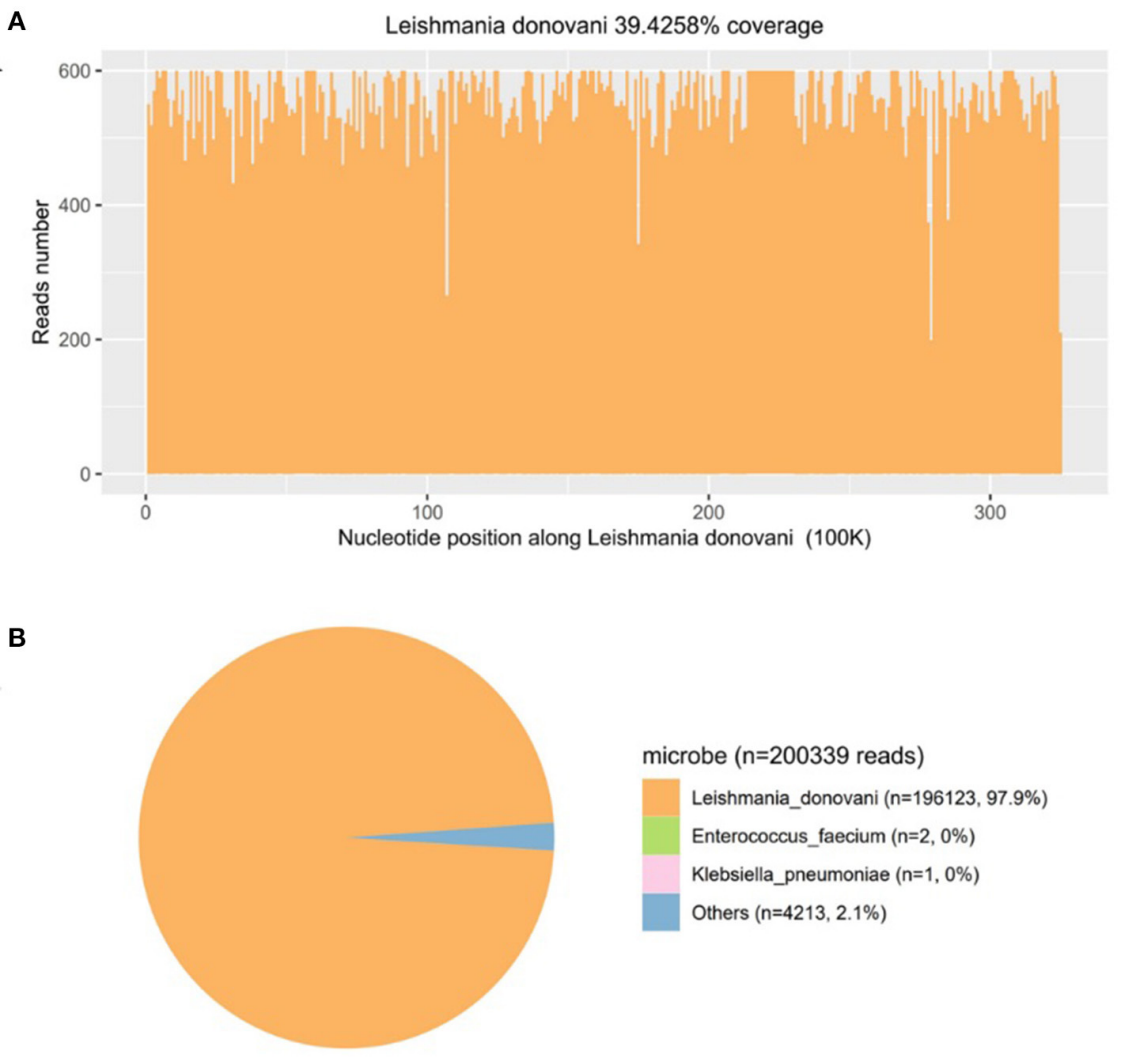
The diagnosis of *Leishmania* infection by metagenomics next-generation sequencing (mNGS). **(A)** Mapping of *Leishmania donovani* reads on the genome. **(B)** Distribution of pathogenic microorganisms reads in the absence of human, others, and unclassified reads.

**Figure 2:**
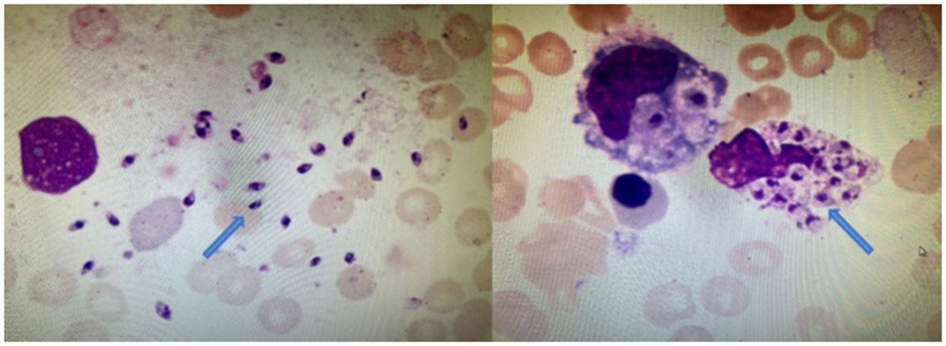
Bone marrow cytology of this patient. Arrowheads show the *Leishmania* spp amastigotes in extracellular and phagocyte, which are oval and 2.9–5.7 × 1.8–4.0 μm in size (Wright's stain, × 1,000).

**Figure 3:**
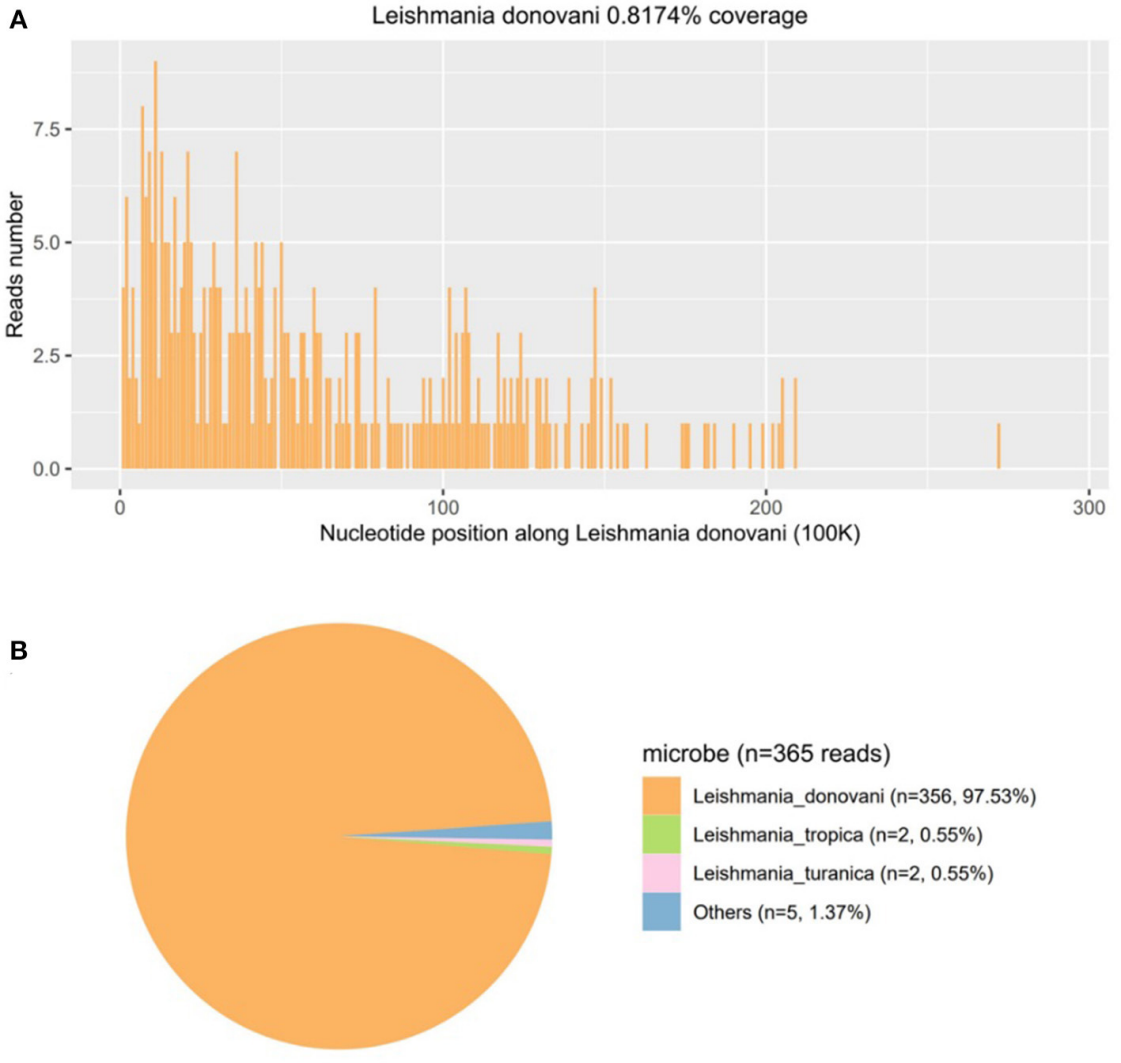
The follow-up diagnosis of *Leishmania* infection after using pentavalent antimonials 10 days by mNGS. **(A)** Mapping of *Leishmania donovani* reads on the genome. **(B)** Distribution of pathogenic microorganisms reads in the absence of human, others, and unclassified reads.

Then, mNGS was carried out in peripheral blood. After DNA was extracted from 200 μl of peripheral blood, the DNA library was built and sequenced on Nextseq 550 platform (Illumina, USA). All human host DNA was filtered out, and the valuable reads were aligned to Microbial Genome Databases (ftp://ftp.ncbi.nlm.nih.gov/genomes/) using BWA. Finally, a number of 196123 special reads of *L. donovani* were detected, and the coverage of the genome and relative abundance of *L. donovani* was 39.43 and 97.9%, respectively (Figure 1), which was indicative of *L. donovani* infection. In addition, 1 special read of *Klebsiella pneumoniae* was detected and defined as a background bacteria. Examination of patients' demographic information revealed the following: ever since birth, he resided in Jiuzhaigou county of Sichuan province, which is known as an endemic leishmaniasis region. Subsequently, microscopy of the bone marrow showed a larger number of *Leishmania* spp amastigotes, while phagocytic phenomena of histiocytes were found in all smears (Figure 2). All bone marrow smears from confirmed leukemia were reviewed, revealing no *Leishmania* spp amastigote in microscopy. Furthermore, RK39 antigens were also positive by immunochromatography. Thus, VL was diagnosed, and sodium stibogluconates were used as an anti-leishmanial drug. Nevertheless, after 10 days of anti-leishmanial therapy, 356 special reads of *L. donovani* were detected in peripheral blood by mNGS (Figure 3), and *Leishmania* spp amastigotes were also observed in the bone marrow. Subsequently, the anti-leishmanial drug Amphotericin B was used as rescue therapy. After completion of therapy, there was no *Leishmania* spp amastigote in the bone marrow. Finally, the child was discharged 56 days after admission. He was subsequently referred to the hematological clinic for leukemia and follow-up. On February 2023, he was readmitted to the hospital for chemotherapy, when mNGS test for peripheral blood was performed, and no reads of *L. donovani* were detected.

## Discussion

In the present study, we described a case of an infant diagnosed with ALL on admission to the hospital with repeated fever and coagulopathy after chemotherapy. A specific pathogen infection was suspected after collecting demographic information and learning about the history of lifelong residence in the forest region in Sichuan province, clinical symptoms, laboratory test results, and treatment history. Subsequently, the diagnosis of VL was definitely confirmed by mNGS. The patient was treated with pentavalent-Sb with adequate dosage and duration, and mNGS detected *356 L. donovani* reads from the patient's plasma sample. Anti-leishmanial drug amphotericin B was subsequently administrated as rescue therapy. To the best of our knowledge, this is a rare report of leishmaniasis diagnosed by mNGS in leukemia, which provides a valuable reference for VL diagnosis and therapy follow-up.

Due to its wide geographic distribution, leishmaniosis constitutes a major public health problem. It is the second most prevalent pathogen among parasitic diseases. Hepatosplenomegaly, anemia, fever, cachexia, and leucopenia are all symptoms of this kind of VL, which can be significantly more dangerous (13). Factors that negatively impair the immune response, such as malnutrition or AIDS, are known to increase the risk of acquiring the infection and result in more severe manifestations (14). Previous studies have reported that VL is a frequent opportunistic infection in HIV-infected immunodeficient individuals that is very rarely found in cancer patients (10, 13). Although immune suppression by treatments or diseases has been rarely described as a risk factor for VL, the most common underlying cause of immunodeficiency in patients with VL are hematological malignancies apart from HIV infection (15). Nonspecific manifestations such as fever, fatigue, hepatosplenomegaly, hepatosplenomegaly, and weight loss may be attributed to malignancy or related treatment, which is difficult to diagnose in patients with tumors (13). Considering the risk of infection, there is a semiquantitative interaction of 2 factors, i.e., epidemiological exposures and the net state of immunosuppression. In this study, the boy resided since birth in an endemic leishmaniasis region of Sichuan province, and anti-leukemia chemotherapy resulted in immunosuppression. Several studies reported a possible association between *Leishmania* infection and cancer (16). Although local immune suppression induced by malignant disorders may promote leishmaniasis development, it is more likely that immunosuppression induced by long-term anti-cancer chemotherapy is responsible for parasite expansion (16).

As *L. donovani* is a specific pathogen that is not commonly present in the environment, patient's epidemiological history and nonspecific manifestations may be easily overlooked. Also, serological or polymerase chain reaction (PCR) reagents for this pathogen are not routinely prepared in the laboratory, which may delay the diagnosis of this infection. Some VL cases have been misdiagnosed as autoimmune hepatitis, ALL, and malignant lymphoma. They can also be asymptomatic, occur in unusual locations, or be clinically or microbiologically refractory (10, 15, 17, 18). In the present case, the symptoms of fever, fatigue, and hepatosplenomegaly were attributed to the ALL or related treatment, and the absence of amastigotes on repeat bone marrow smears to leukemia diagnosis and surveillance, which is why the infection of *L. donovani* was initially ignored. Finally, the infection was definitely confirmed by mNGS as an unknown and refractory infection. The diagnosis of *Leishmania* infection is based on detecting *Leishmania* amastigote, and various diagnostic techniques were used in making the diagnosis. Most studies used combined immunological methods, while others used plot molecular and parasitological tests. Some cases are also challenging to diagnose due to the low parasite load and low levels of antibodies (19). As an unbiased approach to the detection of pathogens, mNGS has allowed crossing the divide from microbial research to diagnostic microbiology, overcoming limitations of current diagnostic tests and allowing for hypothesis-free, culture-independent pathogen detection directly from clinical specimens (20, 21). In addition, for infectious diseases, collecting patient medical history, especially epidemiological history, is very important for diagnosis, as it can help us quickly adopt appropriate detection methods to identify the pathogen. However, in this case, the history of leukemia has its specificity, which has led to the neglect of typical bone marrow microscopy and medical history collection. Nonetheless, future clinical studies are needed to further confirm the value of mNGS in diagnosing *Leishmania* infection.

In summary, VL should be considered a potential opportunistic infection in patients with hematologic malignancies, especially in immunosuppressed patients living in or having visited areas where the disease is endemic. Unbiased mNGS may provide a clinically actionable diagnosis of a specific infectious disease from an uncommon pathogen, eluding conventional testing for weeks after the initial presentation.

## Data availability statement

The original contributions presented in the study are included in the article/supplementary material, further inquiries can be directed to the corresponding author.

## Ethics statement

The studies involving human participants were reviewed and approved by Medicine Ethics Committee of West China Second University Hospital, Sichuan University. Written informed consent to participate in this study was provided by the participants' legal guardian/next of kin. Written informed consent was obtained from the minor(s)' legal guardian/next of kin for the publication of any potentially identifiable images or data included in this article.

## Author contributions

Conception and design: FL. Provision of study materials or patients: LC. Collection and assembly of data: LC, GC, and QY. Data analysis and interpretation: SL, JT, and JD. Manuscript writing and final approval of manuscript: all authors. All authors contributed to the article and approved the submitted version.
